# Supplementary material for: Dual-comb spectroscopy of ammonia formation in non-thermal plasmas
Source: Commun Chem. 2024 May 13;7:110. doi: 10.1038/s42004-024-01190-7 (PMC11091211; doi:10.1038/s42004-024-01190-7)
Supplement: Supplementary file 1 — Description of Additional Supplementary Files [file 42004_2024_1190_MOESM1_ESM.pdf]

Description of Additional Supplementary Files

File name- Supplementary Data 1

File description- Tables of initial parameters and drawing distributions used for each fitted spectrum, along with the mean values and standard deviations that resulted from the Monte Carlo simulation and fitting routine.

File name- Supplementary Data 2

File description- A list of transition frequency adjustments applied to transition frequencies in the HITRAN2020 database.
